# Country data on AMR in Saudi Arabia in the context of community-acquired respiratory tract infections: links between antibiotic susceptibility, local and international antibiotic prescribing guidelines, access to medicine and clinical outcome

**DOI:** 10.1093/jac/dkac219

**Published:** 2022-09-06

**Authors:** Didem Torumkuney, Saeed Dolgum, James van Hasselt, Walid Abdullah, Nergis Keles

**Affiliations:** GlaxoSmithKline, 980 Great West Road, Brentford, Middlesex TW8 9GS, UK; Pediatric Infectious Diseases, Dr. Suliman ALhabib Hospital, Riyadh, Saudi Arabia; GlaxoSmithKline, The Campus, Flushing Meadows, 57 Sloane Street, Bryanston, Gauteng 2021, South Africa; GlaxoSmithKline, Headquarters Business Park, Corniche Road, Jeddah 21544, Saudi Arabia; GlaxoSmithKline, Büyükdere Cad. No: 173, 1. Levent Plaza B Blok 34394 Levent, İstanbul, Türkiye

## Abstract

**Background:**

Antimicrobial resistance (AMR) is one of the biggest threats to global public health. Selection of resistant bacteria is driven by inappropriate use of antibiotics, amongst other factors. COVID-19 may have exacerbated AMR due to unnecessary antibiotic prescribing. Country-level knowledge is needed to understand options for action.

**Objectives:**

To review AMR in Saudi Arabia and initiatives addressing it. Identifying areas where more information is required will provide a call to action to minimize a further rise in AMR within Saudi Arabia and improve patient outcomes.

**Methods:**

National AMR initiatives, antibiotic use and prescribing, and availability of susceptibility data, particularly for the key community-acquired respiratory tract infection (CA-RTI) pathogens *Streptococcus pneumoniae* and *Haemophilus influenzae*, were identified. National and international antibiotic prescribing guidelines commonly used locally for specific CA-RTIs (community-acquired pneumonia, acute otitis media and acute bacterial rhinosinusitis) were also reviewed, plus local antibiotic availability. Insights from a clinician in Saudi Arabia were sought to contextualize this information.

**Conclusions:**

Various initiatives are underway in Saudi Arabia, including a National Action Plan for AMR, which was published in 2017. However, AMR is rising and knowledge about appropriate antibiotic use seems to be lacking among physicians and the general public. Various international guidelines are utilized by clinicians in Saudi Arabia, but a more standardized inclusive approach in developing local guidelines, using up-to-date surveillance data of isolates from community-acquired infections in Saudi Arabia could make management guideline use more locally relevant for clinicians. This would pave the way for a higher level of appropriate antibiotic prescribing and improved adherence. This would, in turn, potentially limit AMR development and improve patient outcomes.

## Introduction

Antimicrobial resistance (AMR) is one of the biggest emerging threats to public health throughout the world,^1^ as described in the introductory paper of this Supplement.^2^ The WHO states that ‘the world urgently needs to change the way it prescribes and uses antibiotics. Even if new medicines are developed, without behaviour change, antibiotic resistance will remain a major threat’.^3^ The first paper in this Supplement included details about the multiple factors which can drive a rise in AMR along with the global initiatives that are in place to address this threat.^2^ Each country and/or region should also play their part through local initiatives.

In order to identify how AMR can be addressed in Saudi Arabia in the future, it is necessary to review what is happening now. In this paper, we present the current situation in Saudi Arabia, determined by using published information (from searching PubMed, Google Scholar and other internet platforms) to ascertain any national initiatives to address AMR, antibiotic use and prescribing, and availability of susceptibility data, in particular for the key community-acquired respiratory tract infection (CA-RTI) pathogens *Streptococcus pneumoniae* and *Haemophilus influenzae*. National and international antibiotic prescribing guidelines for CA-RTIs, specifically community-acquired pneumonia (CAP), acute otitis media (AOM) and acute bacterial rhinosinusitis (ABRS), commonly used by healthcare professionals (HCPs) in Saudi Arabia were also reviewed, along with how these link to local antibiotic availability. Insights from a clinician in Saudi Arabia are included to contextualize this information. In addition, we aimed to identify areas where more information is required and present a call to action to improve clinical outcomes for patients and to minimize further rises in AMR within Saudi Arabia.

## Action Plan

In response to the 2015 initiative from the WHO, the Global Action Plan on Antimicrobial Resistance,^4^ a National Action Plan (NAP) on combatting AMR was published in 2017 in the Kingdom of Saudi Arabia.^5^ The plan introduced a structured approach to the problem of AMR in Saudi Arabia in line with the WHO’s five objectives. The plan recognized the need for an effective ‘One Health’ approach including coordination across different sectors including human and veterinary medicine, agriculture, finance, environment, and consumers.^5^ The current Saudi Arabia NAP status, as reported by the WHO for 2020–21, shows that the Saudi Arabia NAP is being implemented and actively monitored through a monitoring and evaluation framework.^6^

## Antibiotic use and prescribing

In recent decades, the Kingdom of Saudi Arabia has seen an exponential growth in AMR, which is exacerbated by the use of antibiotics without a prescription along with various other factors.^7^ Studies from different areas of Saudi Arabia have revealed poor knowledge and misuse of antibiotics among much of the Saudi public.^7–10^ Many misconceptions regarding the use of antibiotics and lack of awareness of AMR are apparent from surveys of public attitudes.^7,9^ One of the larger studies used structured questionnaires involving 1700 participants from different parts of Saudi Arabia, in the period from June to December 2016. Of the participants, 76.8% had recently used antibiotics with 71.7% taking antibiotics for colds and coughs and 61.8% using antibiotics for treatment of diseases likely to be of viral origin. Most would use antibiotics without prescription. The study found there was a lack of public knowledge about the effects of antibiotic overuse.^11^ In another study in Riyadh, most of the study population (920 participants) was aware of the potential adverse effects of antibiotics and yet nearly 40% had taken non-prescription antibiotics in the last 6 months, with influenza as the most common reason.^12^

Studies have also reported a lack of knowledge and poor practices amongst healthcare professionals (HCPs). A survey of HCPs, including pharmacists and doctors, identified a need for comprehensive training and education about AMR. Insufficient duration of antimicrobial use, using antimicrobials unnecessarily, and use of broad-spectrum antibiotics were reported to be common practices.^13^ A survey of dentists revealed excessive and inappropriate prescription of antibiotics.^14^

Despite the recent introduction of a 2018 law prohibiting dispensing antibiotics without a prescription, there was no significant change in the level of antibiotic use without prescription. It has been recommended that educational programmes and campaigns should be developed to improve public knowledge of AMR and its causes along with increased surveillance of antibiotic dispensing. Increased accessibility for primary health care and provision of health insurance to the population to decrease antibiotic self-medication would also be a recommendation.^15^ For healthcare providers, increasing continuous professional development on appropriate antibiotic prescribing, and providing local antimicrobial guidelines and regular consultation with infectious disease experts on infectious diseases are suggested. More studies about local AMR patterns are necessary, and more efforts are needed to educate clinicians about AMR.^13^

## Surveillance

### National surveillance studies

In Saudi Arabia there are a small number of studies from individual units or hospitals that have provided information on local antibiotic susceptibility rates.^16,17^ While these local data are extremely useful within a specific hospital or city, they cannot provide a wider national picture.

### Global surveillance studies

Currently two ongoing global surveillance studies provide antibiotic susceptibility data for pathogens associated with CA-RTIs in Saudi Arabia.

#### ATLAS

The Antimicrobial Testing Leadership and Surveillance (ATLAS) database is a global AMR surveillance programme which is fully accessible, and which covers susceptibilities of a range of bacterial and fungal pathogens to a bank of antimicrobials with reference to the different breakpoints. ATLAS data is analysed based on CLSI and EUCAST breakpoints. However, the number of isolates tested in ATLAS in Saudi Arabia is currently very low (<10) and hence the results have not been included here.^18^

#### SOAR

The Survey of Antibiotic Resistance (SOAR) is a multinational antibiotic surveillance study, ongoing in an expanding range of countries since 2002. The study aims to collect and make available in published, peer-reviewed papers, antibiotic susceptibility data, specifically for *S. pneumoniae* and *H. influenzae*, which are the most commonly isolated respiratory pathogens in the community.^19^

Key features of the SOAR study are that it focuses only on these pathogens, and that identification and susceptibility testing are performed in an independent centralized laboratory using standardized methodology (CLSI) allowing for comparisons to be made between countries/regions and for the identification of trends over time. SOAR data is analysed based on CLSI, EUCAST dose-specific, and PK/PD breakpoints.

Clinical breakpoints are cut-off MIC values used to classify microorganisms into the clinical categories susceptible (S), intermediate (I) and resistant (R) in order to assist in the prediction of the clinical success or failure of a specific antibiotic.^20^ CLSI and EUCAST define breakpoint values but due to variation in criteria for their definition, there are some differences between CLSI and EUCAST in the clinical breakpoint values for certain bacteria for some antibiotics and this can impact susceptibility interpretation of clinical isolates.^21^ EUCAST breakpoints are dose-specific and use the EMA-approved doses that are included in the Summary of Product Characteristics of an antibiotic. This means that by application of breakpoints for higher doses, the effect of using a raised dose on the clinical efficacy of a particular antibiotic can be predicted. Currently, clinical microbiology laboratories in Saudi Arabia use CLSI breakpoints, however, the international application of the EUCAST breakpoints is expanding,^22^ so it is possible that dose-specific breakpoints could also be applied in Saudi Arabia in future. The EUCAST dose-specific breakpoints can be used retrospectively to calculate the susceptibility of previously collected isolates to show the susceptibility levels that would have been achieved at higher doses.

Use of the EUCAST dose-specific breakpoints shows the effect of increasing the antibiotic dose on the susceptibility of a pathogen, providing additional information so the prescriber can decide if a higher dose would be of benefit. For example, *S. pneumoniae*, the most isolated respiratory pathogen^23,24^ for infections such as CAP, AOM and ABRS, has over time become less susceptible to amoxicillin/clavulanic acid in some countries^25^ since the MICs of some isolates have increased. When treating infections, it is important to be able to eradicate bacterial pathogens with raised MICs in order to optimize clinical outcome, while at the same time minimizing the risk of selecting variants with even higher MICs. This is possible because β-lactams, unlike many other antibiotics, have time-dependent killing properties. Their efficacy depends on the amount of time the drug concentration is present at the site of action. Although increasing the antibiotic concentration at the infection site over a particular concentration will not have any effect on efficacy, the use of higher doses and/or more frequent dosing allows for successful eradication of pathogens with higher MICs because the time above the MIC is increased.^26^

**Figure 1.**
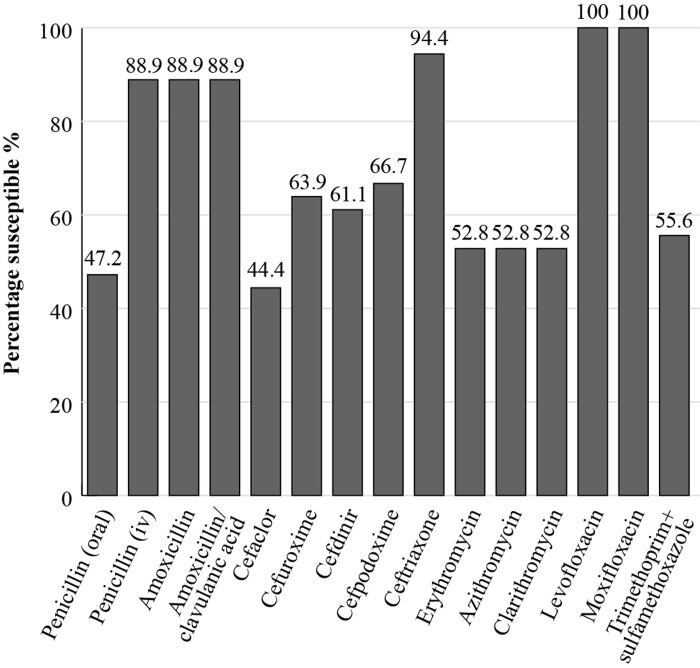
Percentage susceptibility rates based on CLSI breakpoints for antibiotics against *S. pneumoniae* isolates (*n *= 36) collected as part of the SOAR study in Saudi Arabia in 2015–17.

The most recent SOAR 2015–17 results for Saudi Arabia included isolates collected at two sites from outpatients with confirmed CA-RTIs. When applying the CLSI breakpoints, 88.9% of *S. pneumoniae* isolates (*n *= 36) were susceptible to amoxicillin and amoxicillin/clavulanic acid but this fell to 52.8% for the macrolides erythromycin, azithromycin and clarithromycin. For the fluoroquinolones, levofloxacin and moxifloxacin, *S. pneumoniae* susceptibility was 100%.^19^ (Figure 1).

**Figure 2.**
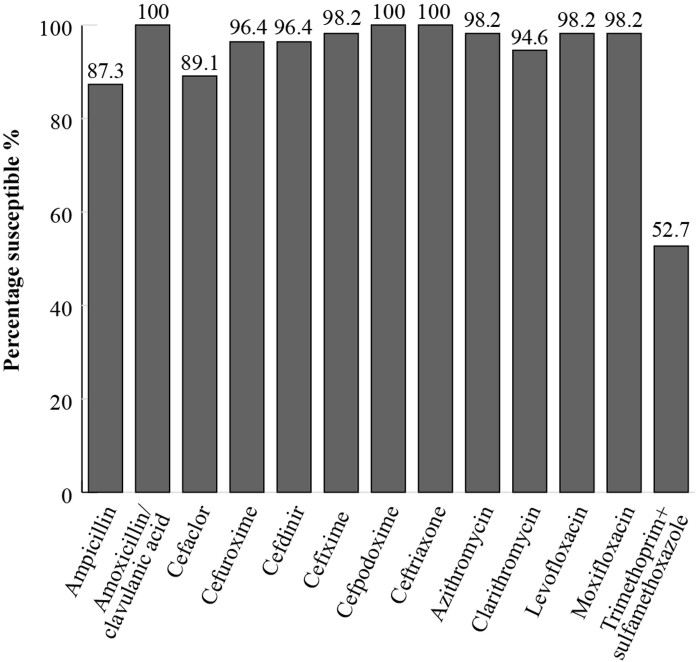
Percentage susceptibility rates based on CLSI breakpoints for antibiotics against all *H. influenzae* isolates (*n *= 55) collected as part of the SOAR study in Saudi Arabia in 2015–17.

For all *H. influenzae* isolates (*n *= 55) from Saudi Arabia collected from patients during the same time period, high susceptibility to amoxicillin/clavulanic acid, cefpodoxime and ceftriaxone (100%) was seen, while susceptibility to ampicillin was 87.3% (Figure 2) by CLSI criteria. The majority of isolates were also susceptible to macrolides (clarithromycin 94.6% and azithromycin 98.2%) and fluoroquinolones (98.2%) using CLSI criteria for *H. influenzae*. All isolates of *H. influenzae* from Saudi Arabia were β-lactamase-negative. Within this population, two were β-lactamase-negative ampicillin-resistant (BLNAR) according to CLSI breakpoints.^19^

#### GLASS

In 2015, the WHO launched the Global Antimicrobial Resistance and Use Surveillance System (GLASS). GLASS is the first global system to collect national AMR data for selected bacterial pathogens that cause common infections. The aim is to monitor the prevalence of AMR among major pathogens in clinical settings^27^ to provide the supporting data required to ensure that countries can design cost-effective, evidence-based AMR response strategies. During the first four years, 91 countries/territories have enrolled in GLASS and data for over two million patients from 66 countries are included.^28^ Pathogens currently included in GLASS-AMR are: *Acinetobacter* spp., *Escherichia coli*, *Klebsiella pneumoniae*, *Neisseria gonorrhoeae*, *Salmonella* spp., *Shigella* spp., *Staphylococcus aureus*, and *S. pneumoniae.* GLASS data is analysed based on CLSI and EUCAST breakpoints. A new and important component is the inclusion of antimicrobial consumption (AMC) surveillance at the national level.^29^ By 2021, the number of countries participating in GLASS had increased to 107, including Saudi Arabia.

## Disease Management Guidelines

For management of the common CA-RTIs, CAP, AOM and ABRS in Saudi Arabia, clinicians use the national antimicrobial therapy guidelines for community- and hospital-acquired infections in adults, plus a range of international guidelines, examples of which are included in Table 1. Most guidelines suggest a first-line antibiotic or antibiotics along with alternative(s) and then a second-line antibiotic or antibiotics, also with an alternative(s). The first-line antibiotic is the recommended first choice that should be prescribed by the clinician following diagnosis of the infection, supported by the criteria defined by the organization or committee. Alternative(s) may be provided for use in particular circumstances. For example, if the first-line antibiotic is a penicillin then alternative suggestions will be for use in the case of penicillin allergy. The second-line antibiotic is for use if the first-line does not achieve the anticipated outcome, and again alternative(s) may be included for use under specific circumstances.

**Table 1.** Examples of local and international antibiotic prescribing guidelines referred to by physicians in Saudi Arabia for the management of community-acquired respiratory tract infections

Local antibiotic prescribing guidelines
Antimicrobial Stewardship Subcommittee of the National Antimicrobial Resistance Committee and the General Administration of Pharmaceutical Care at Ministry of Health. National antimicrobial therapy guidelines for community and hospital-acquired infections in adults (2018)^30^
International antibiotic prescribing guidelines
IDSA 2007: Infectious Diseases Society of America. guidelines on the management of community-acquired pneumonia in adults^31^
IDSA 2011 (Endorsed by AAP): The Management of Community-Acquired Pneumonia in Infants and Children Older Than 3 Months of Age: Clinical Practice Guidelines by the Pediatric Infectious Diseases Society and the Infectious Diseases Society of America^32^
IDSA 2012: IDSA Clinical Practice Guideline for Acute Bacterial Rhinosinusitis in Children and Adults^33^
AAP 2013: American Academy of Pediatrics. The diagnosis and management of acute otitis media^34^
IDSA 2019: Diagnosis and Treatment of Adults with Community-acquired Pneumonia. An Official Clinical Practice Guideline of the American Thoracic Society and Infectious Diseases Society of America^35^

### International antibiotic prescribing guidelines

For the management of CAP in adults and children, the international guidelines referred to by clinicians in Saudi Arabia include those from the Infectious Diseases Society of America (IDSA).^31,32,35^ For example, the first-line recommendation by the IDSA 2019 for treating adult outpatients with CAP but no comorbidities is amoxicillin or doxycycline or a macrolide (if local pneumococcal resistance is <25% or there are risk factors for MRSA or *P. aeruginosa*) but if the patient has comorbidities and CAP, the recommendations include amoxicillin/clavulanic acid, 500 mg/125 mg given three times daily, 875 mg/125 mg or 2000 mg/125 mg both given twice daily, or cephalosporins, in combination with a macrolide or doxycycline, or monotherapy with a respiratory fluoroquinolone.^35^ For the management of AOM, the international guidelines referred to in Saudi Arabia include those from the American Academy of Pediatrics (AAP).^34^ For ABRS in children, the IDSA recommend as initial first-line empirical treatment, amoxicillin/clavulanic acid 45 mg/kg/day twice daily and in adults first-line amoxicillin/clavulanic acid 500 mg/125 mg three times daily or 875 mg/125 mg twice daily.^33^

### National antibiotic prescribing guidelines

Local Saudi Arabian guidelines are available for the management of CAP and ABRS in the National antimicrobial therapy guidelines for community- and hospital-acquired infections in adults (2018).^30^

## Antibiotic availability

In Saudi Arabia, several formulations of amoxicillin/clavulanic acid currently available are mentioned as first-line or second-line recommendations by the RTI management guidelines. According to the IDSA guideline (2019) for CAP, 875 mg/125 mg given twice daily (in combination with a macrolide or doxycycline) is an option for CAP in adult outpatients with comorbidities.^35^

In children who are outpatients with presumed pneumonia, amoxicillin/clavulanic acid (amoxicillin component, 90 mg/kg/day twice daily) is recommended by the IDSA (2011) as an alternative empirical therapy to first-line amoxicillin treatment for CAP.^32^

Saudi Arabia local guidelines for CAP in adults also recommend amoxicillin/clavulanic acid 1 g (1 g/200 mg) IV regimen every 8 h in combination with azithromycin for inpatients (elderly >65 years, non-ICU patients) with comorbidities or recent antibiotic use in the last 3 months, or for ICU adult inpatients with no risk of MRSA or *Pseudomonas*, or with vancomycin IV added for ICU patients at risk of MRSA or pseudomonas.^30^ In AOM, the AAP guidelines are followed, which specifically recommend high dose amoxicillin (80–90 mg/kg/day) given in two divided doses or amoxicillin/clavulanic acid 14:1 formulation (90 mg/kg/day amoxicillin with 6.4 mg clavulanic acid given in two divided doses) for first-line treatment in children with initial or delayed antibiotic treatment and also recommends the high dose amoxicillin/clavulanic acid 14:1 formulation or ceftriaxone for patients who have failed initial antibiotic treatment.^34^

In children with ABRS, the IDSA guidelines recommend amoxicillin/clavulanic acid 45 mg/kg/day (amoxicillin component) given in two doses as initial first-line empirical treatment and 90 mg/kg/day (amoxicillin component) given in two doses as second-line empirical treatment. In adults with ABRS, the IDSA guideline 2012^33^ recommends either 500 mg/125 mg three times daily or 875 mg/125 mg twice daily as initial first-line therapy. The second-line treatment includes amoxicillin/clavulanic acid 2000 mg/125 mg twice daily and this regimen is also an option where there is a risk of antibiotic resistance or failure of initial therapy.^33^

Local guidelines for ABRS recommend oral amoxicillin/clavulanic acid 1000 mg (amoxicillin component) every 12 h as first-line in mild cases. In severe infections requiring hospitalization, amoxicillin/clavulanic acid IV 1 g (1 g/200 mg) every 8 h is recommended.^30^

Substandard poor-quality or falsified antibiotics promote AMR^36^ and the spread of drug-resistant infections. Since poor-quality antibiotics are unlikely to contain the full dose needed to eliminate all of the infecting pathogens this would encourage resistance to develop and allow resistant strains to survive and be transmitted.

The quality of medicines, specifically antibiotics, is an important consideration for countries worldwide. The WHO has launched a Global Surveillance and Monitoring System (GSMS) for substandard and falsified products.^37^ The GSMS aims to work with WHO member states to improve the quality of reporting of substandard and falsified medical products, and, importantly, to ensure the data collected are analysed and used to influence policy, procedure, and processes to protect public health, at the national, regional and global level. Use of substandard or falsified antibiotics not only compromises clinical outcomes but also risks increased AMR. The most recent GSMS summary (2013–17) reported substandard and falsified medicines in 46 member states (not including Saudi Arabia). Antibiotics represent 16.9% of all products reported, second only to malaria drugs (19.6%).^37^

## Local insight

### Clinician expert comment

In recent decades there has been overuse of antibiotics by physicians. Prescribing antibiotics to treat bacterial infections is challenging because the physician must evaluate multiple variables such as the accuracy of the patient history and physical examination, selecting appropriate diagnostic tools such as laboratory tests and radiology, and consideration of the pharmacodynamics and pharmacokinetics of the antibiotic before choosing the appropriate antibiotic for the infection. This requires a high level of knowledge about the infection and the antibiotic.

In Saudi Arabia there are multiple endeavours to establish treatment guidelines for common infections to advise physicians in terms of management and prescribing, such as the Saudi Arabia Ministry of Health protocols for malaria and leishmaniasis. The burden of bacterial resistance is a widely recognized worldwide problem and global institutes and organizations have published guidelines for safe practice and the use of antibiotics. Many guidelines have been produced by institutions such as WHO, CDC, IDSA, and others and these are followed in clinical practice in Saudi Arabia. The factor that would drive the most appropriate management of infectious disease is to choose not international or national susceptibility data but to refer to the local hospital’s antibiogram since there may be significant differences between what is included in international or national guidelines and locally.

Our success in the antimicrobial stewardship programme in Saudi Arabia is based on the recognition of the problem of bacterial resistance and facing the reality of over-prescription of antibiotics by non-infectious disease experts.

## Conclusions

In an era of rising AMR throughout the world, this paper aims to define areas where action is required to tackle AMR by analysing and understanding the current situation within a country or region. In Saudi Arabia a NAP for AMR was introduced in 2017. In this study we have investigated antibiotic use and prescribing, approach to AMR, availability of local susceptibility data, use of international and/or local management guidelines, and how these link to antibiotic availability. To our knowledge this is the first time this information has been reviewed and presented in detail by country.

Over the past few years Saudi Arabia has seen increasing AMR, which is exacerbated by the use of antibiotics without a prescription, even though this is prohibited by a law introduced in 2017. Other factors exacerbating the increase in AMR include inappropriate use and over-prescribing of antibiotics. There is a lack of knowledge and awareness in the population about the risks of antibiotics and AMR, and healthcare providers do not generally adhere to the principles of appropriate antibiotic prescribing, which has led to the overuse of antibiotics and excessive prescription of broad-spectrum agents.

In terms of surveillance there are some small studies from hospitals or individual units that have provided information on local susceptibilities and AMR and Saudi Arabia is also participating in the WHO GLASS-AMR initiative, which should provide useful information in future. Currently the global surveillance study SOAR is key to providing data about the susceptibility of CA-RTI pathogens (*S. pneumoniae* and *H. influenzae*) in Saudi Arabia. The most recent SOAR results reveal a trend towards decreasing susceptibility amongst the common respiratory pathogens for some antibiotic classes such as the macrolides. The fluoroquinolone antibiotics have so far retained activity, although guidelines and regulatory bodies urge caution and the restriction of their use to limited situations due to serious safety concerns.

For the management of the common CA-RTIs, CAP, AOM and ABRS in Saudi Arabia, clinicians make use of some country-specific local antibiotic prescribing guidelines and several international guidelines are also referred to, including those published by the IDSA for CAP and ABRS and the AAP for AOM.

Despite further enforcement of the policy not to dispense antibiotics without a prescription in 2018, there is, however, still misuse of antibiotics in terms of self-medication and the non-prescription use. There is a need for campaigns to increase public knowledge and awareness about the appropriate use of antibiotics. For physicians and other health professionals, an increase in local surveillance studies would provide data to enable the development of regularly updated management guidelines, which would improve clinical outcomes and minimize a further rise in AMR. A more standardized inclusive approach is needed to develop local country-specific guidelines. As described in the Consensus Principles discussed in the introductory paper for this Supplement^2^ and reiterated by the expert clinician’s comments in this paper, appropriate management of infectious disease should be driven not by referring to international susceptibility data and guidelines, but by reference to available local information since there may be significant differences between them.

Local guidelines would be based on up-to-date surveillance data of isolates from community-acquired infections which would make the guideline more locally relevant for clinicians, reiterating the Consensus Principles. This would pave the way for improved adherence and a higher level of appropriate antibiotic prescribing in CA-RTIs which could, in turn, potentially limit AMR development and improve clinical outcomes for patients.
